# Supplementary material for: Are there dedicated neural mechanisms for imitation? A study of grist and mills
Source: PLoS One. 2023 Sep 26;18(9):e0291771. doi: 10.1371/journal.pone.0291771 (PMC10522020; doi:10.1371/journal.pone.0291771)
Supplement: S1 File — (DOCX) [file pone.0291771.s001.docx]

Supplementary Information for:

Are there dedicated neural mechanisms for imitation? A study of grist and mills

Elizabeth Renner, Yishan Xie, Francys Subiaul,

and Antonia F. de C. Hamilton

The tables here include all vertical contrasts (i.e., comparing different demonstration types within tasks) for the execution phase only. For the following simple contrasts, there were no significant clusters: in the cognitive task, Ghost > Hand and Ghost > Text; in the spatial task, Ghost > Hand and Hand > Text. For the Hand > Ghost comparisons, see the manuscript main text.

***Table S1.1*** ***Cognitive Task, Hand > Text:*** *Brain regions, FWE-corrected* p *values, sizes of clusters (k), T values, and MNI coordinates of clusters showing significant activation following Hand vs Text demonstrations (Hand-cognitive > Text-cognitive)*

| **Brain region** | **p(FWE)** | **k** | **T** | **MNI coordinate** | | |
| --- | --- | --- | --- | --- | --- | --- |
|  |  |  |  | x | y | z |
| L fusiform | <0.001 | 296 | 5.77 | −52 | −52 | −8 |
| L superior temporal gyrus |  |  | 5.50 | −48 | −48 | 10 |
| L middle temporal gyrus/fusiform |  |  | 4.88 | −48 | −50 | 2 |

***Table S1.2*** ***Cognitive Task, Text > Hand:*** *Brain regions, FWE-corrected* p *values, sizes of clusters (k), T values, and MNI coordinates of clusters showing significant activation following Text vs Hand demonstrations (Text-cognitive > Hand-cognitive)*

| **Brain region** | **p(FWE)** | **k** | **T** | **MNI coordinate** | | |
| --- | --- | --- | --- | --- | --- | --- |
|  |  |  |  | x | y | z |
| L postcentral gyrus | <0.001 | 786 | 7.73 | −52 | −22 | 40 |
| L precentral gyrus |  |  | 5.78 | −34 | −24 | 56 |
| L postcentral gyrus |  |  | 5.73 | −32 | −36 | 60 |
| R postcentral/  supramarginal gyrus | <0.001 | 149 | 7.63 | 60 | −22 | 28 |
| R postcentral/  supramarginal gyrus |  |  | 4.33 | 64 | −20 | 36 |
| R postcentral/  supramarginal gyrus |  |  | 4.00 | 54 | −22 | 22 |
| L middle occipital gyrus | <0.001 | 231 | 7.10 | −48 | −76 | −6 |
| L middle occipital gyrus |  |  | 7.08 | −48 | −74 | 4 |
| L middle temporal gyrus |  |  | 5.90 | −38 | −68 | 6 |
| R cingulate gyrus | <0.001 | 393 | 6.34 | 4 | −4 | 40 |
| R supplementary motor area |  |  | 5.95 | 4 | −12 | 56 |
| L medial cingulate gyrus |  |  | 5.88 | −6 | −4 | 42 |
| R middle temporal gyrus/ temporal-occipital junction | <0.001 | 187 | 5.47 | 44 | −64 | 10 |
| R middle temporal gyrus/ temporal-occipital junction |  |  | 5.01 | 50 | −72 | −4 |
| R middle temporal gyrus/ temporal-occipital junction |  |  | 4.99 | 46 | −78 | 6 |

***Table S1.3*** ***Cognitive Task, Text > Ghost:*** *Brain regions, FWE-corrected* p *values, sizes of clusters (k), T values, and MNI coordinates of clusters showing significant activation following Text vs Ghost demonstrations (Text-cognitive > Ghost-cognitive)*

| **Brain region** | **p(FWE)** | **k** | **T** | **MNI coordinate** | | |
| --- | --- | --- | --- | --- | --- | --- |
|  |  |  |  | x | y | z |
| L lingual gyrus/ calcarine | 0.014 | 87 | 6.71 | −8 | −92 | −8 |
| L lingual gyrus/ calcarine |  |  | 4.14 | −2 | −86 | −6 |
| L lingual gyrus |  |  | 3.77 | −10 | −94 | 0 |
| R lingual gyrus | <0.001 | 193 | 6.39 | 28 | −58 | −6 |
| R fusiform |  |  | 4.98 | 36 | −50 | −20 |
| R fusiform |  |  | 4.53 | 28 | −46 | −18 |
| L fusiform | <0.001 | 478 | 6.38 | −26 | −72 | −6 |
| L fusiform |  |  | 5.68 | −28 | −66 | −12 |
| L cerebellum |  |  | 5.31 | −24 | −54 | −18 |
| L middle occipital | 0.012 | 91 | 5.49 | −34 | −86 | 16 |
| R lingual | 0.026 | 77 | 4.32 | 14 | −80 | −12 |
| R fusiform |  |  | 4.04 | 34 | −74 | −14 |
| R fusiform |  |  | 3.98 | 26 | −78 | −16 |

***Table S2.1*** ***Spatial Task, Text > Hand:*** *Brain regions, FWE-corrected* p *values, sizes of clusters (k), T values, and MNI coordinates of clusters showing significant activation following Text vs Hand demonstrations (Text-spatial > Hand-spatial)*

| **Brain region** | **p(FWE)** | **k** | **T** | **MNI coordinate** | | |
| --- | --- | --- | --- | --- | --- | --- |
|  |  |  |  | x | y | z |
| R lingual | 0.050 | 70 | 5.85 | 24 | −58 | −10 |
| R fusiform |  |  | 5.50 | 30 | −50 | −10 |
| R fusiform |  |  | 4.43 | 38 | −52 | −12 |

***Table S2.2*** ***Spatial Task, Ghost > Text:*** *Brain regions, FWE-corrected* p *values, sizes of clusters (k), T values, and MNI coordinates of clusters showing significant activation following Ghost vs Text demonstrations (Ghost-spatial > Text-spatial)*

| **Brain region** | **p(FWE)** | **k** | **T** | **MNI coordinate** | | |
| --- | --- | --- | --- | --- | --- | --- |
|  |  |  |  | x | y | z |
| R supplementary motor area | <0.001 | 232 | 6.42 | 2 | −22 | 58 |
| R supplementary motor area |  |  | 5.46 | 12 | −24 | 54 |
| R paracentral lobule |  |  | 5.42 | 2 | −32 | 50 |

***Table S2.3*** ***Spatial Task, Text > Ghost:*** *Brain regions, FWE-corrected* p *values, sizes of clusters (k), T values, and MNI coordinates of clusters showing significant activation following Text vs Ghost demonstrations (Text-spatial > Ghost-spatial)*

| **Brain region** | **p(FWE)** | **k** | **T** | **MNI coordinate** | | |
| --- | --- | --- | --- | --- | --- | --- |
|  |  |  |  | x | y | z |
| R parahippocampal gyrus/ fusiform | <0.001 | 217 | 6.71 | 28 | −60 | −10 |
| R fusiform |  |  | 4.48 | 34 | −52 | −18 |
| R fusiform |  |  | 4.46 | 40 | −58 | −16 |
