# Supplementary material for: Are there dedicated neural mechanisms for imitation? A study of grist and mills
Source: PLoS One. 2023 Sep 26;18(9):e0291771. doi: 10.1371/journal.pone.0291771 (PMC10522020; doi:10.1371/journal.pone.0291771)
Supplement: Attachment — Submitted filename: Response_to_Reviewers_2023-07-18.docx [file pone.0291771.s002.docx]

Dear Editor,

Thank you for the opportunity to revise and improve our manuscript. We detail below how we have addressed the reviewers’ comments; our responses are in blue text. All line references are to the Track-Changes version of the manuscript. As a point of clarification, the data we present in this manuscript have not been previously published; only data from the Demonstration phase (not the Execution phase) were previously analyzed.

Reviewer #1: This paper looks at brain activity during action execution in a delayed imitation task to compare two hypotheses of imitation: that it relies on dedicated brain mechanisms (MNS hypothesis) and that it relies on more general sensorimotor processing (Grist-Mills hypothesis). The results show imitative action execution elicits more occipital brain activity than non-imitative action execution. However, no differences are found in MNS areas.

I think this is an interesting, well-designed, and clearly-written study. I only have a couple of minor comments that I hope may help to further improve the paper:
1. It’s not entirely clear to me why the MNS hypothesis would necessarily predict more brain activity in MNS areas during execution of delayed imitation. Is it because the MNS hypothesis assumes that these areas are not only involved in executing the action, but also in matching the observed action to a motor command? If so, predicting more activity for imitation in the execution phase implies that this matching process has not yet been done by then. Could the authors unpack their prediction a bit more and explain exactly why the MNS hypothesis predicts stronger brain activity during execution?

We have added some detail to the Introduction to address why the MNS hypothesis predicts MNS brain activity during delayed execution (Lines 55-65 and 202-203). In short, the MNS hypothesis derives from the activations of mirror neurons which occur both when an individual views someone else doing an action and when they perform that action themselves. In this hypothesis, the perceptual-motor matching function occurs in the STS (an activation which we did not find in our results).

2. Similarly, it’s not entirely clear to me why the Grist-Mills hypothesis predicts no differences in brain activity. Couldn’t differences in brain activity also reflect differences in input? For example, as the authors suggest in the discussion, it could be that “hand input” and “ghost input” is maintained differently between observation and execution. If true, the mechanisms transforming the visual input to a motor response would still be the same.

The Grist-Mills hypothesis is detailed more thoroughly in Heyes’ 2018 volume *Cognitive Gadgets*. According to this hypothesis, matching vertical associations (formed through experience during development) are excitatory connections between sensory stimuli and (corresponding) motor actions. For example, when someone waves goodbye and you also wave goodbye, the association between seeing a hand wave and waving your own hand is strengthened. The stimuli “can be in any sensory modality and originate from animate or inanimate objects” (p. 126). Furthermore, the “degree of topographic resemblance between observed and executed actions” (p. 127) is never calculated; that is, there is never a topographic matching process (unlike the MNS hypothesis), as the excitatory connections are sufficient to enable imitation.

We agree that the Grist-Mills hypothesis would predict differences in brain activity in the various conditions *during demonstration*; this is precisely when perceptual, attentional, or motivational differences may arise between imitative and non-imitative conditions. However, once perception of the to-be-imitated action has occurred, the brain should execute the social and matched non-social conditions with the same mechanism, as any (e.g., perceptual) biases have already influenced the organism’s processing. We have added some detail about this at Lines 74-80.

3. Why are the execution events modeled as 6s events and not as events with duration equal to the total execution time? If a fixed duration is used, couldn’t execution time influence brain activity?

Execution events were modeled as 6-second events because all execution events, lasted for 6 seconds, even if the sequence was selected more quickly by the participant. That is, if a participant correctly selected all of the target images in <6 seconds, the screen remained blank after the final image disappeared and the ITI did not begin until the full 6 seconds had elapsed. (“Error” trials were not included in the analysis comparing conditions.) Actual participant response times were reported in the previous paper (in Fig. 3); mean correct response times for completed sequences were 3200-4200ms. We have consolidated information about timings in the Experimental Design section (Lines 281-285). The 6-s execution phase duration strikes a balance between allowing sufficient time for participants to complete the sequence, allowing time for the HRF, keeping participants engaged, and performing a sufficient number of trials.

4. The authors write that the absence of activity differences in MNS areas can’t be ascribed to low power because they find differences in other areas. I don’t think this claim is justified, as power of course depends on the effect size. It could also be that there are differences in MNS areas but that these differences are smaller than those in occipital areas.

We agree that interpreting a lack of activation in fMRI is difficult. We have added further elaboration of the possibility of less extensive activations into the Discussion (Lines 418-422) and clarify that even when a lower threshold was used, activations of mirror neuron areas were not found.

Reviewer #2: This paper revisits the question of which brain regions are involved in imitation. Although several fMRI studies addressed this timely question, most suffer from inadequate controls, as discussed in the present paper. Here, the authors contrasted two hypotheses of imitation: the well-known mirror-neuron system hypothesis, implying parieto-frontal regions, and a hypothesis that proposes no differences between social and non-social processes, implying the involvement of the same brain regions in imitation as in non-social performance. They re-analyzed data from their previously published fMRI experiment and their data do not support either hypothesis. The most striking observation was the absence of mirror neuron system imitation-related activations, but null results are difficult to interpret, especially in event-related fMRI. My main concern relates to the interpretation of the occipital activations which the authors attribute to imitation. I believe that more control analyses (and perhaps experiments) are required to make such a conclusion

Main comments.

1. I was wondering whether the occipital activations they observed during the execution phase are not because of a spillover from the activations to the preceding different demonstration conditions (BOLD is a very slow response). Did those activations depend on the time interval between the demonstration and execution phase, being stronger with shorter intervals? If so, then this would support the trivial explanation of spillover. This should be addressed.

Thank you for pointing out the interesting possibility that the occipital activations we found during the execution phase could have been due to a spillover from visual events during the demonstration phase. We now consider this possibility in the manuscript (Lines 460-473). We think that this is an unlikely explanation for the activations during the execution phase, as the locations of the clusters do not correspond to those from the demonstration phase. That is, the demonstration phase clusters are anterior (example coordinates -54 -74 10) to the execution phase clusters (example coordinates -32 -82 0).

2. The “visual” activations appear to include V1 and I find it difficult to believe that V1 would be involved in imitation, except for differences in visual processing between conditions. The same may hold for the more anterior occipital activations (EBA?). Related to this issue, did the viewing (fixation/saccade) patterns during the execution phase differ among the different conditions? Also, were the reaction times the same in the different conditions? Different reaction times relate to differences in exposure to the stimuli and thus differences in occipital activations.

Although occipital lobe is not canonically thought to be involved in imitation, we note in the paper that a number of previous researchers have reported occipital lobe activations during imitative tasks (including Makuuchi 2005 and Caspers et al. 2010).

Viewing patterns were not recorded during the study so we cannot say whether these differed between conditions. Selection of the three target items using the joystick was visually guided (and trials on which errors were made were not included in the analyses).

Reaction times (reported in Fig. 3 in the initial paper) were not different between the conditions for the cognitive task. Reaction times for the spatial task followed the pattern Hand (slowest) > Ghost > Text. Because occipital activations occurred during the execution phase for the cognitive task (Hand > Ghost), in which there were no reaction time differences, it is unlikely that the occipital lobe activations for that task were due to exposure duration differences.

3. The authors contrasted the hand and ghost conditions, ignoring the text condition. However, Figure 2 suggests similar activations between the hand and text condition and also between the text and ghost condition in the occipital areas. They should consider the text condition activations also in the description of the Results and the interpretation.

We focus on the contrasts between the Hand and Ghost conditions because these conditions can address the main question of which brain areas are implicated in executing imitative versus matched non-imitative actions. The Text condition, while important as a control, is not as closely matched to the Hand condition and is therefore not as informative as the Ghost condition.

We had intended to upload a Supplementary Information document with the manuscript, which contains tables with results from the Hand, Ghost, and Text contrasts for both tasks. This was not done due to an oversight, and it has now been uploaded. It is referred to in the manuscript at Lines 369-370.

4. The text condition activations, in comparison to those of the hand condition, make me wonder to what extent the activations in the occipital areas are related to imitation and do not result from confounding, ill-controlled effects of the preceding demonstration phases or during the execution phase (see main points 1 and 2 above). As long as the authors cannot make a convincing case to the contrary, the evidence of occipital (and EBA) being involved in imitation is weak (if not absent), and that should be discussed.

Please see our response to comments 1 and 2. Briefly, we are not the first to report a link between occipital activation and imitation performance. Also, note that in the Discussion we specifically call for caution when interpreting these results (Line 457-459).

5. The absence of mirror neuron system activation is the most interesting finding of this experiment. However, as I mentioned above, null results in event-related fMRI are difficult to interpret, and that should be acknowledged.

We agree that the reasons for null results can be manifold and have added a clarification about this to the text at Lines 418-422.
